# *CIPK11*: a calcineurin B-like protein-interacting protein kinase from *Nitraria tangutorum*, confers tolerance to salt and drought in *Arabidopsis*

**DOI:** 10.1186/s12870-021-02878-x

**Published:** 2021-03-01

**Authors:** Lu Lu, Xinying Chen, Pengkai Wang, Ye Lu, Jingbo Zhang, Xiuyan Yang, Tielong Cheng, Jisen Shi, Jinhui Chen

**Affiliations:** 1 Key Laboratory of Forest Genetics & Biotechnology of Ministry of Education, Co-Innovation Center for Sustainable Forestry in Southern China, Nanjing Forestry University, Nanjing, 210037 China; 2 College of Biology and the Environment, Nanjing Forestry University, Nanjing, 210037 China; 3 Experimental Center of Desert Forestry, Chinese Academy of Forestry, Dengkou, Inner Mongolia China; 4 Research Center of Saline and Alkali Land of National Forestry and Grassland Administration, China Academy of Forestry, Beijing, 100091 China

**Keywords:** Halophyte, *Nitraria tangutorum*, *CIPK11*, Salt stress, Drought stress

## Abstract

**Background:**

The *CIPKs* are a group of plant-specific Ser/Thr protein kinases acting in response to calcium signaling, which plays an important role in the physiological and developmental adaptation of plants to adverse environments. However, the functions of halophyte-derived *CIPK*s are still poorly understood, that limits a potential application of *CIPKs* from halophytes for improving the tolerance of glycophytes to abiotic stresses.

**Results:**

In this study, we characterized the *NtCIPK11* gene from the halophyte *Nitraria tangutorum* and subsequently analyzed its role in salt and drought stress tolerance, using *Arabidopsis* as a transgenic model system. *NtCIPK11* expression was upregulated in *N. tangutorum* root, stem and blade tissues after salt or drought treatment. Overexpressing *NtCIPK11* in *Arabidopsis* improved seed germination on medium containing different levels of NaCl. Moreover, the transgenic plants grew more vigorously under salt stress and developed longer roots under salt or drought conditions than the WT plants. Furthermore, *NtCIPK11* overexpression altered the transcription of genes encoding key enzymes involved in proline metabolism in *Arabidopsis* exposed to salinity, however, which genes showed a relatively weak expression in the transgenic *Arabidopsis* undergoing mannitol treatment, a situation that mimics drought stress. Besides, the proline significantly accumulated in *NtCIPK11*-overexpressing plants compared with WT under NaCl treatment, but that was not observed in the transgenic plants under drought stress caused by mannitol application.

**Conclusions:**

We conclude that *NtCIPK11* promotes plant growth and mitigates damage associated with salt stress by regulating the expression of genes controlling proline accumulation. These results extend our understanding on the function of halophyte-derived *CIPK* genes and suggest that *NtCIPK11* can serve as a candidate gene for improving the salt and drought tolerance of glycophytes through genetic engineering.

**Supplementary Information:**

The online version contains supplementary material available at 10.1186/s12870-021-02878-x.

## Background

Soil salinity and drought are critical environmental threats to plant development that limit plant growth by negatively affecting the availability, transportation, and partitioning of nutrients and water. These effects are threatening to decline crop productivity worldwide and increase the pace of soil desertification, further affecting the ecological balance [1]. Therefore, understanding halophyte plant tolerance to salt and drought stress is critical for sustaining agricultural productivity by breeding new stress-tolerant plants that may cope with abiotic stresses [2]. *N. tangutorum* belongs to the family Nitrariaceae *Nitraria* in Sapindales, which is widely distributed in northwestern China [3–6]. *N. tangutorum* is a desert halophyte adapted to severe drought and high salinity, and generally grows in arid or semiarid regions with high salinity [7, 8]. Moreover, this species can efficiently alleviate the degree of soil salinity and fix moving sand, thus playing an important ecological role in environmental balance [8, 9]. Previous studies have shown that *Nitraria* may adapt to abiotic stress conditions through increasing antioxidant enzyme activities, proline accumulation, level of soluble carbohydrates and reducing the intracellular Na^+^ / K^+^ ratio [7, 10–13]. However, the molecular mechanisms underlying the physiological adaptability of *N. tangutorum* to various stresses need further study [14–16].

To perceive salinity and drought stress, plants have evolved various stress sensors, signaling pathways, transcription factors and promoters to elicit the necessary responses by altering their metabolism, growth and/or development [17, 18]. Ca^2+^ acts as an ubiquitous messenger in various signal transduction networks to induce specific cellular responses, such as responses to signals of abiotic stress [19, 20]. Previous studies have identified proteins able to sense Ca^2+^ levels, including *CaM*, *CDPK* and *CBL*. *CBL*s function through interacting with *CIPK*s to activate specific targets and transduce signals [21, 22]. *CIPK*s contain a highly conserved N-terminal kinase domain with a putative activation loop and a unique C-terminal regulatory region with a conserved NAF amino-acid motif that have been found to promote stress tolerance by regulating various physiological responses [23–25]. Overexpression of *OsCIPK12* improved rice tolerance to cold, drought, and salt stress by inducing the accumulation of proline and soluble sugars [26]. *CaCIPK6* from chickpea has been shown to mediate auxin transport to regulate the salt tolerance of tobacco seedlings [27]. Overexpression of *BrCIPK1* enhanced abiotic stress tolerance by increasing proline biosynthesis in rice [28]. In addition, *CIPKs* may regulate the activity of the ROS scavengers POD, SOD and CAT to reduce the content of H_2_O_2_ and MDA, and to improve stress tolerance [29, 30] or they may control ion and water homeostasis to improve salt tolerance [31, 32]. These findings have continuously revealed the importance of *CIPK*s in regulating physiological factors that may improve plant stress tolerance.

Here, we identified a novel member of the *CIPK* gene family from *N. tangutorum*, *NtCIPK11*, and describe its role in the molecular regulation of salt and drought tolerance. We found that *NtCIPK11* was induced in root, stem and leaf tissues by 500 mM NaCl or 200 mM mannitol, with transcripts preferentially accumulating in leaves. To further explore how *NtCIPK11* might function molecularly, we overexpressed it in *Arabidopsis*. The transgenic plants showed a higher germination rate and better growth than the WT plants after NaCl or mannitol treatment. In addition, we found that genes involved in glutamate-derived proline biosynthesis [33–35], were regulated in transgenic plants. Besides, the proline accumulation was found to be significantly higher in the transgenic plants than that of WT seedlings. On the contrary, the H_2_O_2_ content showed a less level in *NtCIPK11*-overexpressing plants than WT. Our data show that *NtCIPK11* is able to regulate the proline accumulation through mediating the expression of key genes of specific biochemical processes in *Arabidopsis*, thereby increasing tolerance of plants dealing with abiotic stresses.

## Results

### *N. tangutorum* physiologically responded to salt treatment

**Fig. 1:**
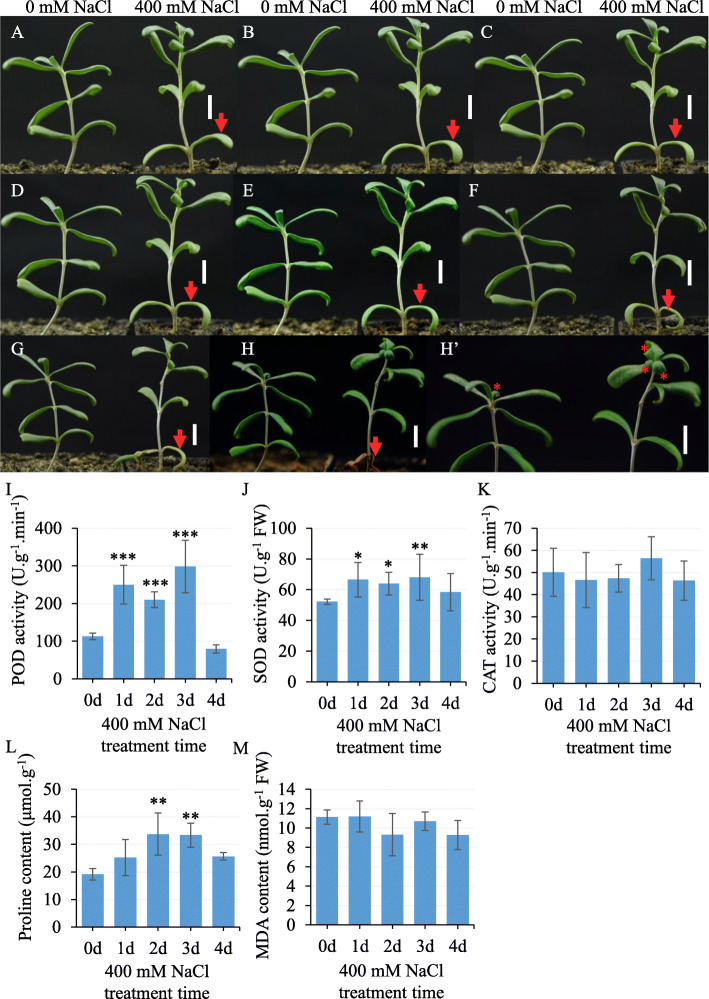
*N. tangutorum* morphologically and biochemically responded to NaCl stress. **a**-**h** Morphology of *N. tangutorum* during salt treatment: 0 mM NaCl (Left) and 400 mM NaCl (right) treated plants for 0 day (**a**), 1 day (**b**), 2 days (**c**), 3 days (**d**), 4 days (**e**) and 8 days (**f**); the appearance of the plants after the 8-day treatment as described above and 1-day re-watering (**g**) and 10-day re-watering (**h** and **h′**) with tap water; red arrowheads indicate withering leaves; red stars indicate new leaves; scale bar: 1 cm. **i**-**m** Effect of NaCl stress on biochemical parameters: activities of POD (**i**), SOD (**j**), and CAT (**k**), proline content (**l**), and MDA content (**m**) in the *N. tangutorum* leaves. The data represent means ± SD of three biological replicates; statistical analyses were performed with one-way ANOVA test with LSD multiple comparisons, ‘*’ *p* < 0.05, ‘**’ *p* < 0.01, ‘***’ *p* < 0.001

As a halophyte with adaptability in a salt environment, *N. tangutorum* has been the focus of studies designed and implemented to investigate the mechanism of salt tolerance using biochemical methods [7, 10, 12] and molecular biology techniques [14, 15, 36]. To better understand the salt tolerance, we observed the growth morphology of *N. tangutorum* upon 400 mM NaCl treatment (Fig. 1). The seedlings watered with tap water showed unchanging growth state for 18 days (0 mM NaCl treated plants in Fig. 1a-h). However, the plants treated with 400 mM NaCl exhibited dynamic change in appearance. The bottom leaves gradually withered and turned yellow with treatment extension. After one week, the seedlings under salt stress conditions were significantly different from the untreated seedlings, especially the bottom leaves (Fig. 1a-f). However, plants treated with salt for one week recovered when tap water was used for another 10 days and displayed more vigorous growth than the untreated plants. More new leaves appeared at the tip of the salt-treated seedlings (Fig. 1g, h and h’). To further study the physiological mechanism of salt tolerance, the activity of antioxidant enzymes POD, SOD and CAT, was tested in plants after 400 mM NaCl treatment (Fig. 1i-k). The results showed that the activity of these antioxidant enzymes was differentially affected by salinity. The POD and SOD activity increased significantly at a 400 mM salinity level on the first day of treatment (Fig. 1i and j). CAT did not positively respond to salt treatment in our experiment (Fig. 1k). Furthermore, we found that free proline, generally thought to have a positive role in plants responses to environmental stresses, such as drought and salinity [37, 38], significantly accumulated in *N. tangutorum* after salt treatment (Fig. 1l). In addition, the MDA content, which indicates the integrity of the membrane [39], was slightly changed during the salt treatment (Fig. 1m). Thus, these data taken together suggest that *N. tangutorum* significantly increased the activity of some antioxidant enzymes and the proline content to protect the cell membrane from being drastically affected by salinity stress under our experimental conditions.

### *NtCIPK11* identification and bioinformatics analysis

**Fig. 2:**
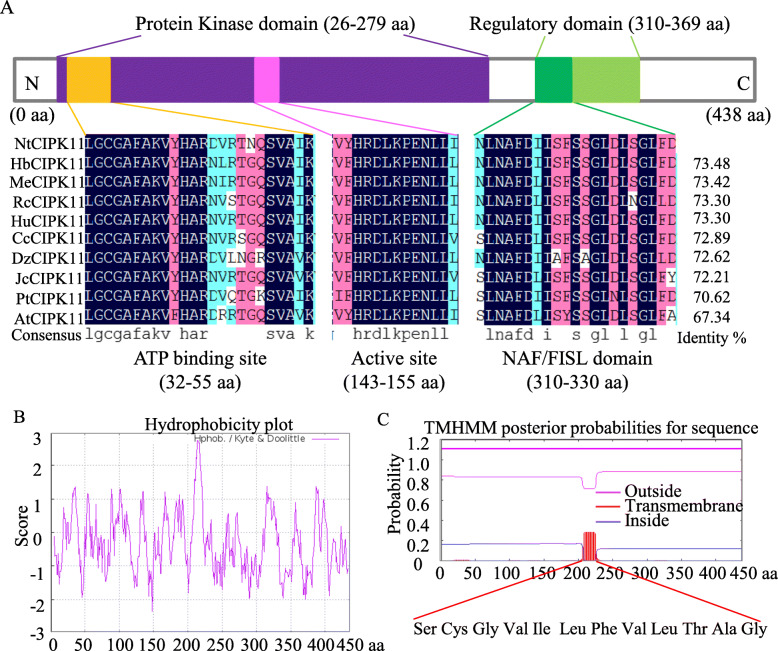
Multiple alignment and domain prediction of NtCIPK11. **a** Multiple alignments for the conserved domains of CIPK11 orthologs from *N. tangutorum* and other species; the borders of the protein domain were predicted by InterProScan online software. **b** Hydrophobicity plot of NtCIPK11. **c** Predicted transmembrane domain of NtCIPK11

**Fig. 3:**
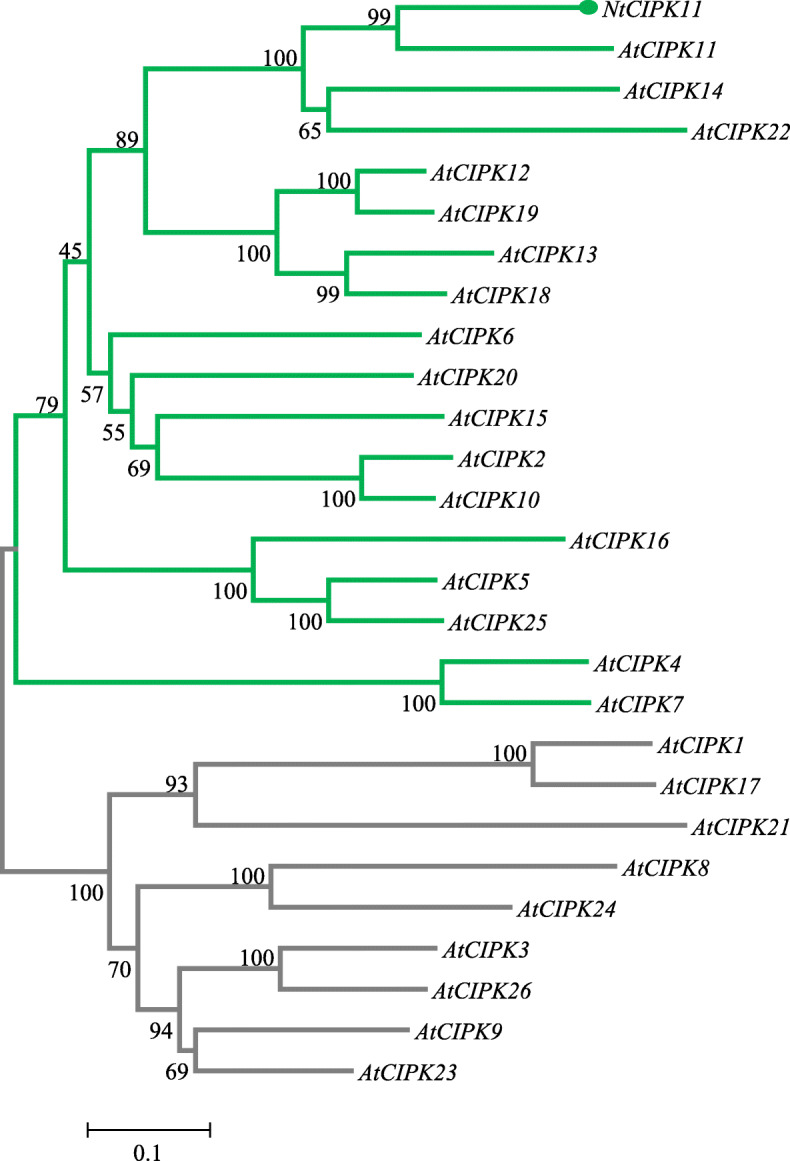
Phylogenetic analysis of NtCIPK11 with *Arabidopsis* CIPKs. The grey branch represents the subgroup of *CIPKs* with introns. The green branch represents the clusters without introns

A large number of plant genes that show a response to different stresses have been previously identified as potential resources for genetic engineering. However, most of these candidate genes were isolated from glycophytes, which possess a relatively poor ability to tolerate environmental stresses [40]. Thus the molecular information from halophytes that can be used to analyze the mechanisms of stress tolerance is limited. As a consequence, *N. tangutorum* was selected for functional gene exploration in our study. We used 5′ and 3′ RACE to determine the complete cDNA nucleotide sequence of the novel gene and found that it is 1677 bp in length, with a 236 bp 5’UTR and a 127 bp 3’UTR. The coding region is 1314 bp long and encodes a 438 amino acid polypeptide with a calculated molecular mass of 49.4126 kDa. BLASTP searches and multiple alignment analyses showed that the deduced protein sequence of this clone displayed a high identity with CIPK orthologs in other species (Fig. 2a). The protein sequence showed 73.48% identity with *Hevea brasiliensis* CIPK11 (XP_021639925.1), 72.62% identity with CIPK11 (XP_006431996.1) of *Citrus clementina* and 67.34% identity with AtCIPK11 (AAK16686.1) of *Arabidopsis thaliana* (Fig. 2a). Similar to its homologues, this deduced protein possesses an N-terminal serine/threonine protein kinase domain (26–279 aa) with an ATP-binding site, an active site and a C-terminal regulatory domain (310–369 aa) with a CBL-interacting NAF/FISL module (Fig. 2a), motifs that are highly conserved in the CIPK family. A hydrophobicity blot and transmembrane domain prediction indicated that the most hydrophobic segment of NtCIPK11 is located between amino acid residues 210 and 221 (Fig. 2b and c). In addition, a phylogenetic analysis of the *N. tangutorum* CIPK protein and 26 *Arabidopsis thaliana* CIPK proteins showed that the novel halophyte CIPK clusters as a sister branch of AtCIPK11 to the intron-free subgroup [41]; hence we referred to it as *N. tangutorum CIPK11* (*NtCIPK11*) (Fig. 3).

### *NtCIPK11* in *N. tangutorum* positively responded to salt treatment

**Fig. 4:**
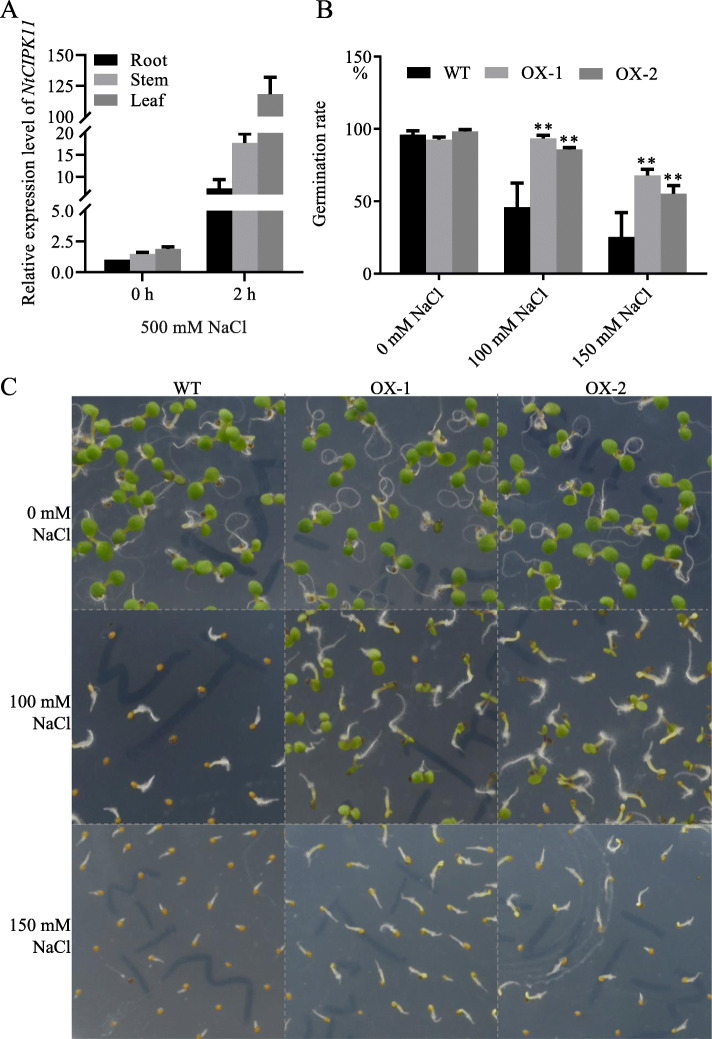
*NtCIPK11* responded to salt stress in *N. tangutorum* and *Arabidopsis*. **a** *NtCIPK11* transcription increased after 500 mM NaCl treatment of *N. tangutorum*. **b** Germination rate of WT and *NtCIPK11* overexpressing seeds. **c** Growth of the seeds germinated on medium with different salt contents. Three biological replicates and three technical replicates were conducted. The data represent the means ± SD from three biological replicates. ‘**’ *p* < 0.01

To study whether *NtCIPK11* expression is regulated by salt in *Nitraria*, we treated seedlings with 500 mM NaCl for a duration of two hours. The qPCR expression profiling showed that untreated *NtCIPK11* was expressed in the roots, stems and leaves, with the latter two tissues expressing 1.4- and 1.8-fold higher levels than the roots (Fig. 4a). After treatment with 500 mM NaCl, we found that the *NtCIPK11* transcript level increased 7-fold in roots, 17-fold in stems and up to 118-fold in leaves compared to the expression level in untreated roots. This finding shows that *NtCIPK11* transcripts accumulate preferentially in leaf tissues after salt treatment (Fig. 4a).

### *NtCIPK11* overexpression led to improved salt resistance in *Arabidopsis*

**Fig. 5:**
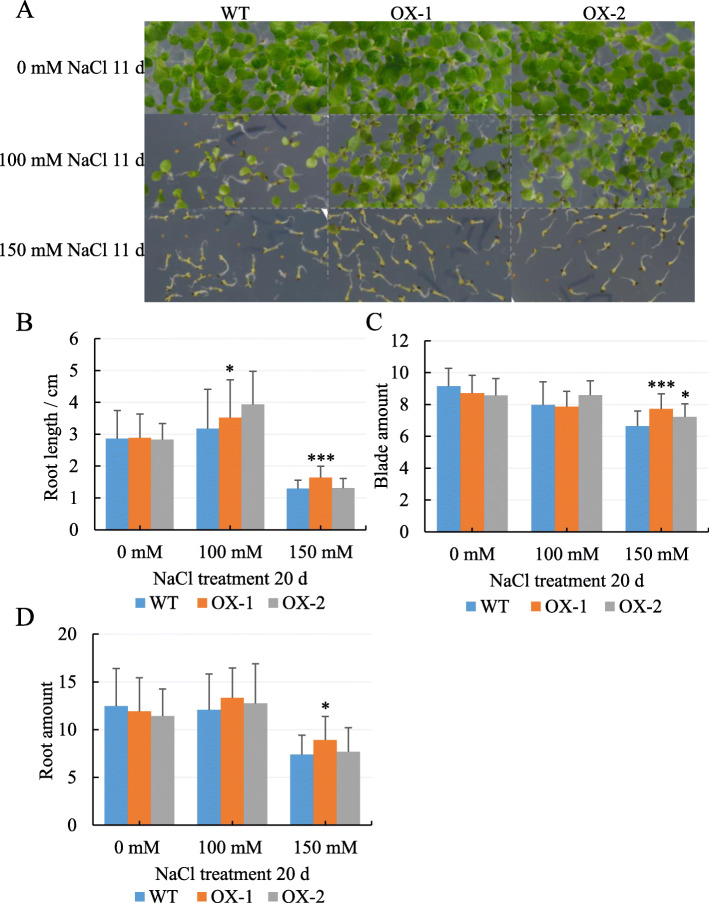
*NtCIPK11* overexpression promoted the growth of *Arabidopsis* under salt conditions. **a** Phenotype of the WT and *NtCIPK11*-overexpressing plants under different salt conditions for 11 days; (**b**) root length; (**c**) blades and (**d**) roots of WT and transgenic plants 20 days post-germination on medium containing different levels of salt. The data represent means ± SD from three biological replicates, and the statistics analyses were performed with one-way ANOVA test, ‘*’ *p* < 0.05, ‘**’ *p* < 0.01

To investigate how *NtCIPK11* acts molecularly, we cloned and overexpressed the gene in *Arabidopsis*. The seeds of transgenic *Arabidopsis* plants showed a 95.66% germination rate on average, close to that of WT seeds (96.05%) on ½ MS medium without salt; however, the *NtCIPK11*-transformed seeds showed 88% or 57% germination rates, respectively, after 5 days of 100 mM NaCl or 150 mM NaCl treatment, approximately twice as high as the WT germination rates of 45% and 25% respectively under the same salt conditions (Fig. 4b and c). After 20 days, the *NtCIPK11*-overexpressing plants showed longer roots (Fig. 5b) and a higher number of leaves (Fig. 5a and c) and roots (Fig. 5d) than the WT plants, with the difference particularly large between the plants treated with 150 mM NaCl-treated medium. Therefore, we concluded that *NtCIPK11* overexpression significantly promoted the seed germination and induced the salt tolerance of *Arabidopsis*.

### Overexpression of *NtCIPK11* altered the transcription pattern of genes involved in proline metabolism and accumulation

**Fig. 6:**
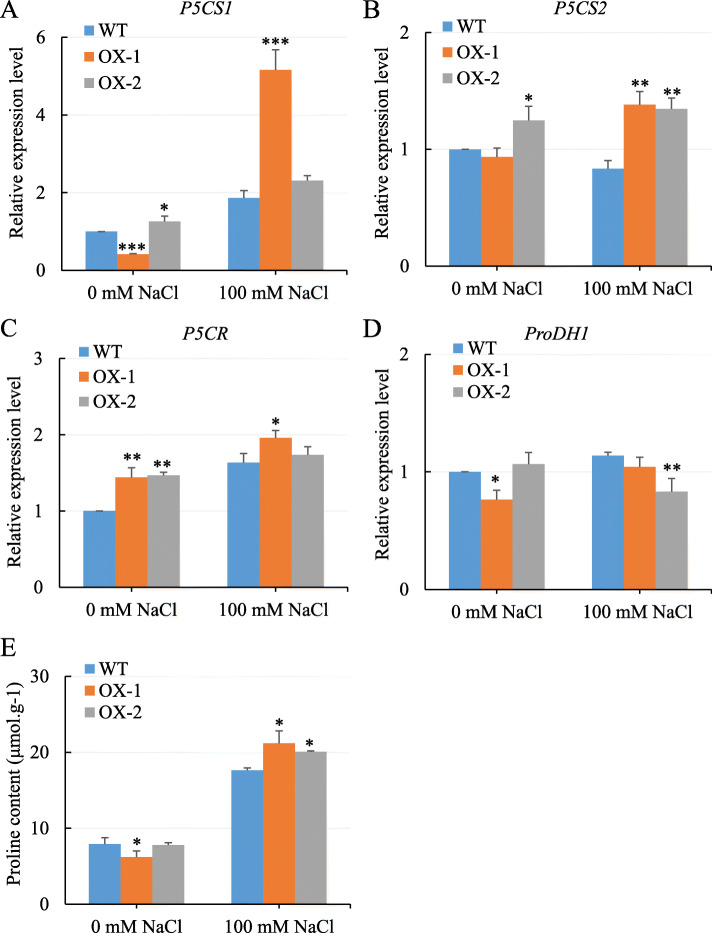
*NtCIPK11* induced the transcription of genes involved in proline metabolism under salt treatment. **a**-**c** Expression levels of proline synthetase genes *P5CS1* (**a**), *P5CS2* (**b**), and *P5CR* (**c**). **d** Expression level of the proline catabolism gene *ProDH1*. **e** Proline content in one-week-old WT and transgenic seedlings germination on the medium containing 0 mM NaCl or 100 mM NaCl. The data represents means ± SD of three replicates and the statistical analyses were performed with one-way ANOVA test, ‘*’ *p* < 0.05, ‘**’ *p* < 0.01

In plants, proline has been reported to accumulate after exposure to various stresses, including salt, drought and cold stress [42]. As shown in previous research, *CIPK* overexpression promoted proline accumulation and improved the tolerance of plants exposed to cold and drought stress [43]. To determine the potential mechanism of how ectopic expression of *NtCIPK11* increases salt tolerance, four key genes of proline metabolism, *P5CS1*, *P5CS2*, *P5CR* [34] and *ProDH1* [35], in WT and transgenic plants were measured via qPCR. As shown in Fig. 6, the genes related to proline synthesis had significantly higher expression levels in the *NtCIPK11-*overexpressing plants than they did in the WT plants under the salt stress conditions (Fig. 6a-c). However, *ProDH1*, which regulates proline catabolism, had a lower expression level in the transgenic plants than in the WT plants (Fig. 6d). Importantly, the proline content was significantly higher in the transgenic seedlings than that of in the WT plants under 100 mM NaCl treatment (Fig. 6e). Besides, H_2_O_2_ staining was observed as light brown in the root of transgenic plants especially in the OX-1 seedlings, but dark brown in WT plants under 100 mM NaCl treatment (Fig. S1). These results showed that *NtCIPK11* overexpression affected the expression of proline metabolism-related genes and proline accumulation, which might mediate the reduction of ROS production to mitigate the damage in plants exposed to salt stress.

### *NtCIPK11* positively responded to drought treatment in *N. tangutorum*

**Fig. 7:**
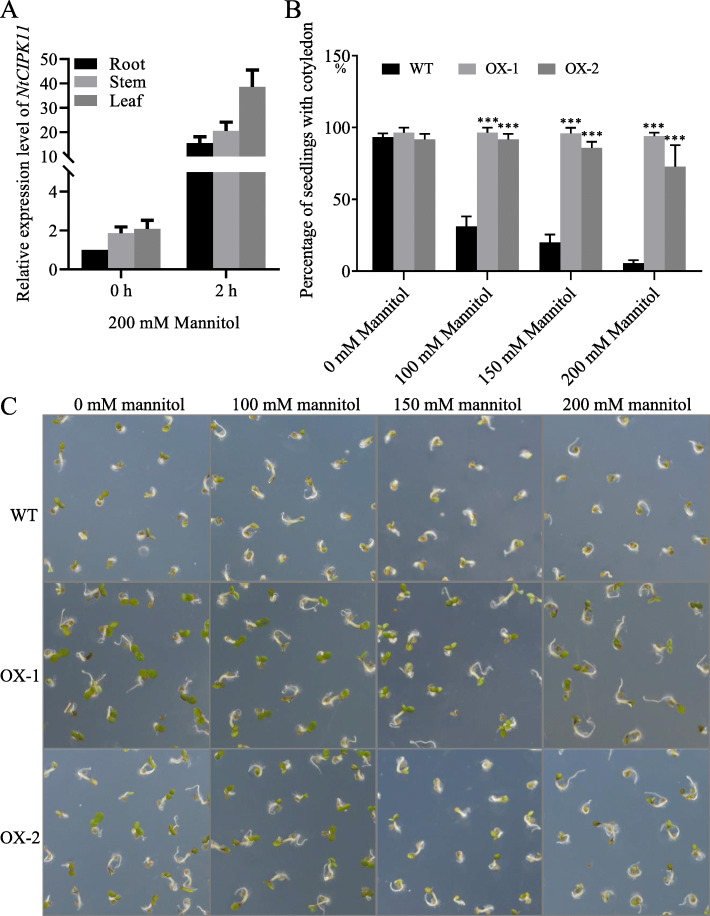
*NtCIPK11* responded to drought stress in *Nitraria* and *Arabidopsis*. **a** Transcription analysis of *NtCIPK11* in *N. tangutorum* after salt treatment. **b** The percentage of *Arabidopsis* seedlings with two cotyledons. **c** Morphology of seedling germination of WT and transgenic *Arabidopsis* plants under increasing mannitol treatment. The data represent means ± SD, three biological replicates, with ANOVA test used for the statistical analyses, ‘*’ *p* < 0.05, ‘**’ p < 0.01, ‘***’ *p* < 0.001

To investigate the function of *NtCIPK11* in drought tolerance, we simulated drought stress by treating plants with 200 mM mannitol for 2 h and observed how *NtCIPK11* expression changed. We found that *NtCIPK11* transcript levels increased dramatically after mannitol treatment, but to a slightly lesser extent than they did upon salt treatment, increasing 15-, 20- and 38-fold in root, stem and leaf tissues, respectively (Fig. 7a). Taken together, these results show that in response to at least two kinds of abiotic stresses, salt and drought stress, *NtCIPK11* expression is increased.

### Overexpression of *NtCIPK11* enhanced the development of *Arabidopsis* seedlings under drought stress

To study how *NtCIPK11* affects the drought stress response, seeds of transgenic *Arabidopsis* and those of WT plants were sown on ½ MS-agar plates containing various concentrations of mannitol. Compared to the seedlings exposed to the salt treatment, the seed germination of both WT and transgenic plants was not affected by the mannitol treatment (Fig. 7c). However, we found that WT seedlings developed more slowly than those of the transgenic plants, as indicated by the percentage of seedlings that formed two cotyledons 4 days post-germination (Fig. 7b and c). Adding mannitol to the ½ MS medium caused a high number of WT seeds to undergo arrested development, with 31%, 20% and 5% of the seedlings reaching the two-cotyledon stage at concentrations of 100 mM, 150 mM and 200 mM mannitol, respectively (Fig. 7b). In contrast, as many as 91%, 80% and 70% of the *NtCIPK11*-transformed seeds developed two cotyledons (Fig. 7b). Therefore, these results showed that *NtCIPK11* can promote seedling development under drought stress conditions at an early stage of plant growth.

### Overexpression of *NtCIPK11* promoted *Arabidopsis* root growth under drought stress

**Fig. 8:**
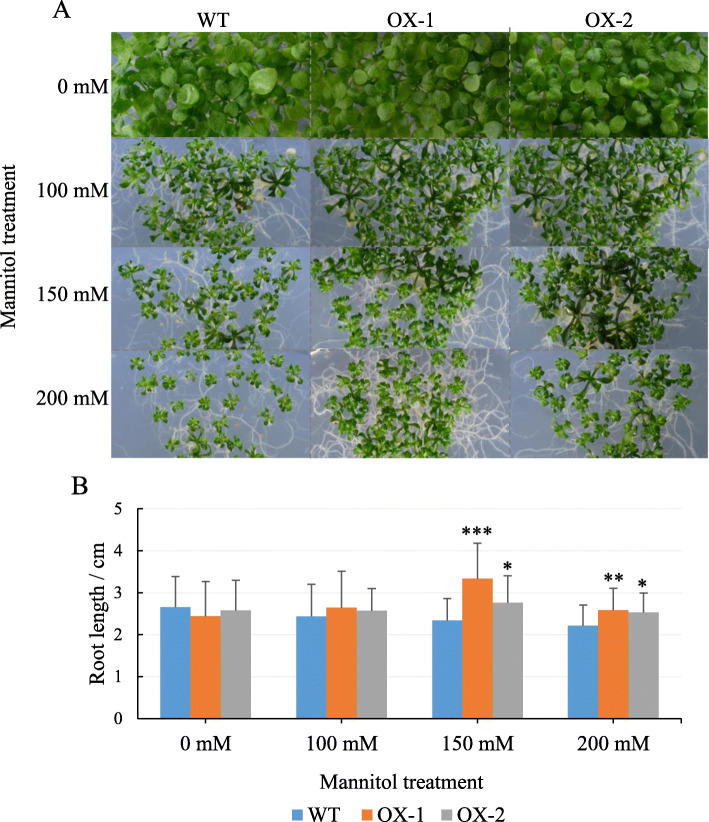
*NtCIPK11*-overexpressing *Arabidopsis* plants developed a longer root. **a** WT and transgenic plants on medium with different concentrations of mannitol. **b** The length of the primary root in the transgenic plants and WT. The data represent the means ± SD of three biological replicates, with ANOVA test used for the statistical analyses, ‘*’ *p* < 0.05, ‘**’ *p* < 0.01

**Fig. 9:**
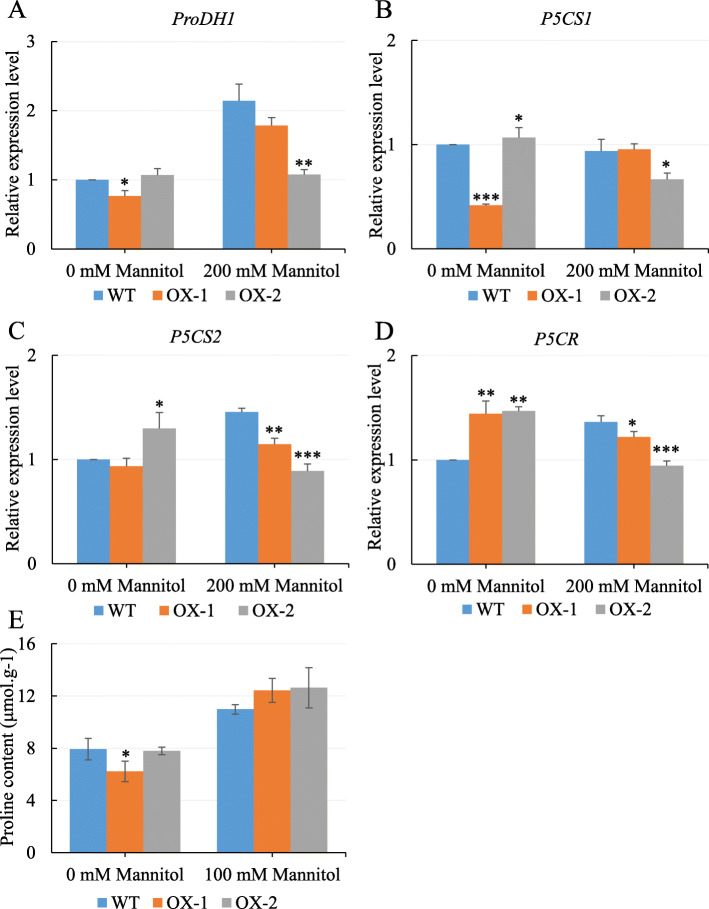
*NtCIPK11* influenced the expression of genes controlling proline metabolism under drought stress. **a** Transcription of *ProDH1*, (**b**) *P5CS1*, (**c**) *P5CS2* and (**d**) *P5CR* after 200 mM mannitol treatment. **e** Proline content in one-week-old WT and transgenic seedlings germinating on the medium containing 0 mM Mannitol or 200 mM Mannitol. The data represent the means ± SD; three technical and biological replicates were conducted; one-way ANOVA test was used for the *p*-value calculations, ‘*’ *p* < 0.05, ‘**’ *p* < 0.01, ‘***’ *p* < 0.001

To further study the function of *NtCIPK11* during drought treatment, we observed plant growth for 20 days on medium containing different concentrations of mannitol. The *NtCIPK11*-overexpressing plants showed better growth than the WT plants after mannitol treatment (Fig. 8a). The transgenic lines developed a longer primary root than the WT line, especially after treatment with 150 mM or 200 mM mannitol (Fig. 8a and b). To determine whether *NtCIPK11* functions like its orthologs to regulate the expression of genes related to proline-mediated drought tolerance, the transcripts of four genes (*ProDH1*, *P5CS1*, *P5CS2*, and *P5CR*) were measured by qPCR, and the results were compared to the transcription patterns of the WT and *NtCIPK11*-overexpressing plants. We found that *ProDH1* transcription in the transgenic plants was lower than it was in the WT plants after mannitol treatment, which indicates a positive effect on proline accumulation (Fig. 9a). Nevertheless, the proline synthesis genes exhibited a different expression pattern compared to the genes under salt treatment in *Arabidopsis* (Fig. 9b-d). At the same time, we observed that the proline content was increased in both WT and transgenic plants under mannitol treatment (Fig. 9e). The proline content of transgenic seedlings was higher than that in the WT. However, that result did not show a significant difference between WT and *NtCIPK11*-overexpressing seedlings (Fig. 9e). These results suggest that *NtCIPK11* is involved in drought and salt stress signaling by influencing the expression of proline metabolism regulators and proline accumulation but to different degrees.

## Discussion

Salt and drought stress are major environmental factors that threaten agricultural productivity and ecological balance. As a result of natural selection and adaptation to a stressful environment, halophytes have evolved specific and diverse regulatory mechanisms for high stress tolerance, that lead to a significant plasticity in environmental adaptation [44, 45]. Thus, the basic machinery of halophytes for adaptation to harsh stresses deserves further research. In addition, understanding the genetics of halophyte responses to a variety of stress conditions is critical for developing transgenic treatment strategies [46–48].

In our study, we reached three major conclusions on how the *NtCIPK11* gene isolated from *N. tangutorum* increases the salt tolerance of *Arabidopsis*. First, the overexpression of *NtCIPK11* in *Arabidopsis* resulted in a significantly higher seed germination rate after NaCl treatment (Fig. 4b and c). Second, the transgenic plants grew better than the WT plants during salt treatment (Fig. 5). Third, the *NtCIPK11* overexpression caused a higher proline accumulation than WT plants under salt stress (Fig. 6). These results revealed the function of this novel gene from the halophyte on salt tolerance were very consistent with the findings of previous studies [49]. The mechanism for salt tolerance induction through *CIPK*s have been previously identified: *CIPK*s mediate the expression of genes encoding various transporters important for ion homeostasis [49–51], increase the amount of antioxidant metabolites [52], or promote the accumulation of compatible osmolytes such as soluble sugars and proline [53, 54] under salt stress conditions. Moreover, the previous report discussed the active response of *N. tangutorum P5CS* to salt stress [55], which helps to explain the proline accumulation in *N. tangutorum* we observed in our study. Therefore, we hypothesized that *NtCIPK11* functions through proline accumulation to protect plants under high salinity conditions. As a result, the key genes regulating proline levels were found to be differentially expressed in *NtCIPK11*-overexpressing plants and WT plants under salt stress. The genes modulating proline synthesis were upregulated (Fig. 6a-c); in contrast, gene regulating proline catabolism was downregulated (Fig. 6d), which would improve proline content in theory. The increased proline content further supported the differential expression of genes related to proline metabolism under salt treatment.

The importance of this study is shown by the finding that the transcription levels of specific genes regulating proline content in transgenic plants were significantly different from those in WT plants when NaCl was applied. However, we found that *P5CS1* and *P5CS2* significantly upregulated under salt stress were downregulated in transgenic plant OX-1 and upregulated in OX-2 under the normal condition (without NaCl treatment). Moreover, we also observed a less proline content of OX-1 than that of WT plants under control condition, that further supported the low expression of genes involved in proline synthesis in transgenic seedlings. The possible reason for this result was supposed to be related with the identity of CBL-CIPK complex. CBL sensor proteins need to bind Ca^2+^ and activate the downstream targets CIPKs, thereby regulating the specific biochemical processes [56]. In other words, a relatively low concentration of Ca^2+^ under normal condition might be the regulatory factor on the activation of CBL-CIPK signaling network and other downstream target genes. Thus, the different concentration of Ca^2+^ in WT and two transgenic plants could be the potential reason for the odd expression of *P5CS1* and *P5CS2* under control condition. However, although the low expression of genes caused a low proline accumulation in the transgenic seedlings, the plants did not suffer from abiotic stresses, thus showing without any significant change on the appearance compared with WT plants under normal condition (Figs. 5 and 8).

On the contrary, abiotic stresses inducing a high concentration of Ca^2+^ signal requires a high activity of CBL-CIPK signal network to activate downstream target genes for responses to stresses. The particular genes involved in abiotic stresses would function as the regulatory element in Ca^2+^ transduction. In our study, the overexpression of *NtCIPK11* regulated the differential expression of genes related to proline metabolism under salt stress. Moreover, decrease of ROS in the transgenic *Arabidopsis* further explained the positive function of *CIPK11* from *N. tangutorum* on plants dealing with salt stress (Fig. S1). Our investigation shared partial points with the research of *CIPK*s from rice [43]. Ectopic expression of rice *OsCIPK03* and *OsCIPK12* led to a significant accumulation of proline under cold and drought stress conditions [43]. Thus, we suggest that our halophyte-derived *NtCIPK11* enhances salt tolerance by inducing gene expression to enhance the proline accumulation in plants exposed to salt stress.

Proline has been proposed to act as a compatible osmolyte [57, 58], a reactive oxygen species scavenger [59], and a protectant of macromolecules such as enzymes and cellular structures [42, 60], thus affecting plant adaptability to stress. Salt-induced *NtCIPK11* regulation of genes associated with proline accumulation led to our speculation about the capacity of this gene to cope with drought stress. Surprisingly, the drought conditions simulated by 100 mM, 150 mM, and 200 mM mannitol application did not affect seed germination but limited seedling development (Fig. 7c). Correspondingly, proline synthetases were not upregulated in transgenic plants (Fig. 9b-d). However, the transcript level of the enzyme leading to proline degradation was higher in the WT seedlings than in the *NtCIPK11*-overexpressing seedlings (Fig. 9a). These results seem to indicate that *NtCIPK11* has no dramatic effect on the proline level under drought stress. The possible reason for this outcome might be attributed to the different strategies of plants in adapting to salinity and drought. Although stress regulators have multiple functions, they have a low probability of showing the same capacity in response to different stresses. For example, *Arabidopsis CIPK11* has been reported as a negative regulator of the drought stress response by controlling the expression of the transcription factor Di19–3, a gene reported to be involved in the abiotic stress response [61]. *NtCIPK11* overexpression led to the advanced development of seedlings after germination under drought stress caused by mannitol treatment (Fig. 8). Thus, we suggest that *NtCIPK11* can promote drought tolerance but the mechanism may not involve proline accumulation under drought conditions.

## Conclusion

In summary, we identified a stress-responsive gene *NtCIPK11* from halophyte *N. tangutorum*. Ectopic expression of *NtCIPK11* promoted the seed germination and seedling growth of *Arabidopsis* upon salt treatment. Moreover, the overexpression of *NtCIPK11* caused a different transcription of genes related to proline metabolism and a relatively high proline accumulation in the seedlings treated with NaCl. Although the transcription patterns of the proline synthesis genes were differentially regulated under mannitol treatment compared to the genes in seedlings under salt stress, *NtCIPK11* overexpression still induced a higher tolerance of the seedlings to drought stress. Hence, it is concluded that *NtCIPK11* is a novel halophyte gene that plays positive roles in plant responses to salt and drought. The novel halophyte-derived gene identified may be used as a candidate gene in molecular breeding of commercial plants to obtain better stress tolerance.

## Methods

### Plant culture and treatment

#### *N. tangutorum*

Seeds of *N. tangutorum* were harvested from the Experimental Center for Desert Forestry of Chinese Academy of Forestry at Dengkou Inner Mongolia in China. The research institution providing the seeds of *N. tangutorum* has a cooperative relationship with Nanjing Forestry University. The author Jingbo Zhang, a professional researcher on genus *Nitraria*, undertook the formal identification of *N. tangutorum*, who harvested the seeds totally complying with the institutional guidelines. We were unfortunately unable to find a voucher specimen of *N. tangutorum* deposited in any publicly available herbarium.

For successful germination, *N. tangutorum* seeds were kept in sand with a relative water content of 7% at 4 °C for eight weeks. The seeds germinated in pots containing a mixture of soil and sand (1:1) in a chamber with 55% to 60% relative humidity, 26 °C ~ 28 °C, and a 16-h light/8-h dark light regime. Two-month-old seedlings has been used for biochemical parameter assays and qPCR analysis.

#### *Arabidopsis thaliana*

The seeds of *Arabidopsis thaliana* (Columbia ecotype) wild type used in this study were kindly provided by Prof. Thomas Laux (Signalling Research Centres BIOSS and CIBSS, Faculty of Biology, University of Freiburg, Germany). Transgenic *Arabidopsis* plants were obtained using the floral dip method [62]. To generate seeds for phenotypic analyses, *NtCIPK11* overexpressing plants were screened until homozygous seeds were obtained. Each experiment was performed in triplicate, with at least 120 seeds of each genotype. *Arabidopsis* seeds were surface sterilized and sown on ½ MS containing different concentrations of NaCl or mannitol, and then cultured in a growth chamber at 23 °C using a 16-h-light/8-h-dark cycle. Four days post-germination, the germination rate and seedling development of the plants were observed; subsequently, the growth state was analyzed 20 days post-germination. *Arabidopsis* seedlings at 20 days post germination were immediately frozen in liquid nitrogen and stored at − 80 °C for qPCR detection.

### Biochemical parameter assay

Two-month-old *N. tangutorum* seedlings were watered with 400 mM NaCl for morphological observation and biochemical parameter assays. The analyses of enzyme activity, proline content and MDA content were conducted following the methods of Janmohammadi et al. (2012) [63] and Zhou et al. (2014) [64]. One-week-old *Arabidopsis* seedlings were harvested for the measurement of proline content. Three technical and biological replicates were performed for each biochemical parameter test. One-way ANOVA test combined with LSD multiple comparisons was used for statistical analysis.

### *NtCIPK11* gene cloning

Total RNA was extracted from the leaves of *N. tangutorum* seedlings using a total RNA purification kit (Norgen, Thorold, ON, Canada), followed by removal of genomic DNA contaminant using DNase I (TaKaRa, Japan). Ultraviolet spectrophotometry was used to quantify the total RNA concentration and gel electrophoresis was used to evaluate its integrity. Double-stranded cDNA was synthesized by reverse transcriptase according to the manufacturer’s instructions (Invitrogen, Carlsbad, USA). Degenerate primers for *NtCIPK11* fragment isolation were designed based on the poplar *CIPK* homeodomain. Primers used for *NtCIPK11* fragment isolation are listed in Supplementary Table 1. The full length of *NtCIPK11* sequence was cloned by 5′ and 3′ RACE using the primers listed in Supplementary Table 2, as indicated in the SMARTerTM RACE cDNA amplification kit user manual (BD Bioscience Clontech, USA). The complete coding sequence of *NtCIPK11* was obtained from cDNA based on the assembled RACE sequences, using the primers listed in Supplementary Table 3.

### *NtCIPK11* sequence analysis

*NtCIPK11* orthologues from other species were searched with NCBI BLASTP. The molecular mass of *NtCIPK11* was predicted by the online software package ExPASy (https://web.expasy.org/cgi-bin/protparam/). Multiple sequence alignments of *NtCIPK11* and its orthologs were performed using DNAMAN 6.0. The feature motifs and domains in *NtCIPK11* were predicted using InterProScan online software (http://www.ebi.ac.uk/InterProScan/). The accession numbers of the sequences and species used for the alignment are listed in Supplementary Table 4. Hydrophobic analysis and transmembrane domain prediction of the NtCIPK11 protein were conducted using the ProtScale tool (http://ca.expasy.org/tools/protscale.html) and the TMHMM Server 2.0 (http://www.cbs.dtu.dk/services/TMHMM/). Phylogenetic analysis was performed with amino acid sequences of *NtCIPK11* and 26 *CIPKs* from *Arabidopsis* using Mega 6 by the NJ method with 1000 bootstrap replications and the JTT model. The accession numbers of the sequences used for the phylogenetic tree are listed in Supplementary Table 5.

### Quantitative real-time PCR analyses

To confirm the response of *NtCIPK11* to salt and drought stress, qPCR was performed using total RNA from the root, stem and leaf tissues of two-month-old *N.tangutorum* seedlings treated with 500 mM NaCl or 200 mM mannitol for 2 h. *NtCIPK11*-overexpressing and WT *Arabidopsis* germinated on medium with 100 mM NaCl and 200 mM mannitol were used for the transcription analysis of proline-related genes. Total RNA was reverse transcribed as mentioned previously. qPCR was carried out using a SYBR-Green PCR Master Mix on a LightCycler®480 real-time PCR detection system (Roche, Basel, Switzerland) according the manufacturer’s instructions. The expression levels of the target genes were normalized by the transcription of the housekeeping gene actin in *Nitraria* [65] and *UBQ10* in *Arabidopsis* [66]. Three technical replicates for each experiment was performed in three biological replicates. The primers used for the qPCR analyses were designed with Primer3 http://frodo.wi.mit.edu/). The sequences of the specific primers for each gene are listed in Supplementary Table 6.

### Detection of H_2_O_2_ accumulation

One-week-old *Arabidopsis* seedlings have been used for H_2_O_2_ accumulation analysis. Eighteen plants from each line (WT and two transgenic lines) were immersed with DAB (Sigma-Aldrich, catalog number: D12384) staining solution for the detection of H_2_O_2_ [67]. Morphology of seedlings staining for four hours was imaged using a Leica M165FC microscope.

## Supplementary Information

**Additional file 1. **Primers and accession information of the genes used in our study: **Supplementary Table 1**. Primers for isolation of the *NtCIPK11* fragment; **Supplementary Table 2**. Primers for RACE; **Supplementary Table 3**. Primers for complete coding region of *NtCIPK11* gene; **Supplementary Table 4**. CIPKs from other species for the conserved domain analysis; **Supplementary Table 5**. *CIPK* family genes in *Arabidopsis* for the phylogenic analysis; **Supplementary Table 6**. Primers for the qPCR analyses.

**Additional file 2: Fig. S1. Detection of H**_**2**_**O**_**2**_ **accumulation in Arabidopsis.** H_2_O_2_ staining with DAB for one-week-old Arabidopsis seedlings (left: WT; middle: OX-1; right: OX-2) cultured under the control condition (A), 100 mM NaCl treatment (B) and 200 mM Mannitol treatment (C). Blue arrows show the light brown root; white arrow shows the dark brown root. Scale bar: 0.2 cm.

## Data Availability

*NtCIPK11* sequence data has been submitted to the NCBI database with accession no. MW014363. All the other data supporting the results of this article are included within the paper and its supplementary file as figures or tables.

## Abbreviations

ROS: Reactive oxygen species
POD: Peroxidase
SOD: Superoxide dismutase
CAT: Catalase
MDA: Malondialdehyde
P5CS: Pyrroline-5-carboxylate synthetase
P5CR: Pyrroline-5-carboxylate reductase gene
ProDH1: Proline dehydrogenase gene
qPCR: Quantitative real-time PCR
JTT: Jones-Taylor-Thornton
UBQ10: Ubiquitin 10
CaM: Calmodulin
CDPK: Calcium-dependent protein kinase
CBL: Calcineurin B-like proteins
CIPK: CBL-interacting protein kinases
NAF: Asn-Ala-Phe
FISL: Phe-Ile-Ser-Leu
RACE: Rapid amplification of cDNA ends
WT: Wild type
OX: Overexpression
DAB: 3,3-diaminobenzidine

## References

[1] Aarati K, Shital D, Raffaella G, Asaph A, Trijatmiko KR, Nayelli MM, Arjun K, Nataraja KN, Makarla U, Andy P (2007). Improvement of water use efficiency in rice by expression of HARDY, an Arabidopsis drought and salt tolerance gene. Proc Natl Acad Sci U S A.

[2] Mahajan S, Tuteja N (2005). Cold, salinity and drought stresses: An overview. Archives of Biochemistry & Biophysics.

[3] Du J, Yan P, Dong Y (2010). Phenological response of Nitraria tangutorum to climate change in Minqin County, Gansu Province, Northwest China. Int J Biometeorol.

[4] Chase MW, Reveal JL (2010). A phylogenetic classification of the land plants to accompany APG III. Bot J Linn Soc.

[5] Lu L, Li X, Hao Z, Yang L, Zhang J, Peng Y, Xu H, Lu Y, Zhang J, Shi J (2018). Phylogenetic studies and comparative chloroplast genome analyses elucidate the basal position of halophyte Nitraria sibirica (Nitrariaceae) in the Sapindales. Mitochondrial DNA.

[6] Bremer B, Bremer K, Chase MWF, Michael F, Reveal JL, Soltis DE, Soltis PS, Stevens PF, Anderberg AA, Moore MJ, Olmstead RG (2009). An update of the angiosperm phylogeny group classification for the orders and families of flowering plants: APG III. Bot J Linn Soc.

[7] Yang Y, Wei X, Shi R, Fan Q, An L (2010). Salinity-induced physiological modification in the callus from halophyte Nitraria tangutorum Bobr. J Plant Growth Regul.

[8] Zhao K, Hai F, Ungar IA (2002). Survey of halophyte species in China. Plant Sci.

[9] Kang JJ, Yue LJ, Wang SM, Zhao WZ, Bao AK (2016). Na compound fertilizer stimulates growth and alleviates water deficit in the succulent xerophyte Nitraria tangutorum (Bobr) after breaking seed dormancy. Soil Sci Plant Nutr.

[10] Yang Y, Shi R, Wei X, Fan Q, An L (2010). Effect of salinity on antioxidant enzymes in calli of the halophyte Nitraria tangutorum Bobr. Plant Cell Tissue & Organ Culture.

[11] Wang L, Ma YK, Li NN, Zhang WB, Mao HP, Lin XF (2016). Isolation and characterization of a tonoplast Na+/H+ antiporter from the halophyte Nitraria sibirica. Biol Plant.

[12] Yang F, Ding F, Duan X, Zhang J, Li X, Yang Y (2014). ROS generation and proline metabolism in calli of halophyte Nitraria tangutorum Bobr. To sodium nitroprusside treatment. Protoplasma.

[13] Yang Y, Yang F, Li X, Shi R, Lu J (2013). Signal regulation of proline metabolism in callus of the halophyte Nitraria tangutorum Bobr. Grown under salinity stress. Plant Cell Tissue & Organ Culture.

[14] Zheng L, Dang Z, Li H, Zhang H, Wu S, Wang Y, Zheng L, Dang Z, Li H, Zhang H (2014). Isolation and characterization of a Δ1-pyrroline-5-carboxylate synthetase (NtP5CS) from Nitraria tangutorum Bobr. And functional comparison with its Arabidopsis homologue. Mol Biol Rep.

[15] Zheng LL, Gao Z, Wang J, Zhang HR, Wang YC (2014). Molecular cloning and functional characterization of a novel CBL-interacting protein kinase NtCIPK2 in the halophyte Nitraria tangutorum. Gmr.

[16] Cheng T, Chen J, Zhang J, Shi S, Zhou Y, Lu L, Wang P, Jiang Z, Yang J, Zhang S (2015). Physiological and proteomic analyses of leaves from the halophyte Tangut Nitraria reveals diverse response pathways critical for high salinity tolerance. Front Plant Sci.

[17] Shinozaki K, Yamaguchishinozaki K (2007). Gene networks involved in drought stress response and tolerance. Jexpbot.

[18] Golldack D, Li C, Mohan H, Probst N (2014). Tolerance to drought and salt stress in plants: unraveling the signaling networks. Front Plant Sci.

[19] Albrecht V, Weinl S, Blazevic D, Dangelo C, Batistic O, Kolukisaoglu U, Bock R, Schulz B, Harter K, Kudla J (2003). The calcium sensor CBL1 integrates plant responses to abiotic stresses. Plant J.

[20] Bertorello AM, Zhu JK (2009). SIK1/SOS2 networks: decoding sodium signals via calcium-responsive protein kinase pathways. Pfluegers Archiv.

[21] Luan S, Lan W, Lee SC (2009). Potassium nutrition, sodium toxicity, and calcium signaling: connections through the CBL-CIPK network. Curr Opin Plant Biol.

[22] Tang R-J, Zhao F-G, Garcia VJ, Kleist TJ, Yang L, Zhang H-X, Luan S (2015). Tonoplast CBL-CIPK calcium signaling network regulates magnesium homeostasis in Arabidopsis. Proc Natl Acad Sci U S A.

[23] Kyung-Nam K, Hwa CY, Grant JJ, Pandey GK, Sheng L (2003). CIPK3, a calcium sensor-associated protein kinase that regulates abscisic acid and cold signal transduction in Arabidopsis. Plant Cell.

[24] Kudla J, Xu Q, Harter K, Gruissem W, Luan S (1999). Genes for calcineurin B-like proteins in Arabidopsis are differentially regulated by stress signals. Proc Natl Acad Sci U S A.

[25] Albrecht V, Ritz O, Linder S, Harter K, Kudla J (2014). The NAF domain defines a novel protein-protein interaction module conserved in Ca2+−regulated kinases. EMBO J.

[26] Yong X, Yuemin H, Lizhong X (2007). Characterization of stress-responsive CIPK genes in rice for stress tolerance improvement. Plant Physiol.

[27] Tripathi V, Parasuraman B, Laxmi A, Chattopadhyay D (2009). CIPK6, a CBL-interacting protein kinase is required for development and salt tolerance in plants. Plant J.

[28] Abdula SE, Lee H-J, Ryu H, Kang KK, Nou I, Sorrells ME, Cho Y-G (2016). Overexpression of BrCIPK1 gene enhances abiotic stress tolerance by increasing Proline biosynthesis in Rice. Plant Mol Biol Report.

[29] Deng X, Wei HU, Wei S, Zhou S, Zhang F, Han J, Chen L, Yin LI, Feng J, Fang B (2013). TaCIPK29,a CBL-interacting protein kinase gene from wheat,confers salt stress tolerance in transgenic tobacco. PLoS One.

[30] Wang Y, Sun T, Li T, Wang M, Yang G, He G (2016). A CBL-interacting protein kinase TaCIPK2 confers drought tolerance in transgenic tobacco plants through regulating the Stomatal movement. PLoS One.

[31] Pandey GK, Poonam K, Amarjeet S, Leonie S, Amita P, Yadav AK, Indu T, Sanyal SK, Beom-Gi K, Sung-Chul L (2015). Calcineurin B-like protein-interacting protein kinase CIPK21 regulates osmotic and salt stress responses in Arabidopsis. Plant Physiol.

[32] Chen X, Huang Q, Fan Z, Bo W, Wang J, Zheng J (2014). ZmCIPK21, a maize CBL-interacting kinase, enhances salt stress tolerance in Arabidopsis thaliana. Int J Mol Sci.

[33] Strizhov N, Abrahám E, Okrész L, Blickling S, Zilberstein A, Schell J, Koncz C, Szabados L (1997). Differential expression of two P5CS genes controlling proline accumulation during salt-stress requires ABA and is regulated by ABA1, ABI1 and AXR2 in Arabidopsis. The Plant journal.

[34] Jung Y, Park J, Choi Y, Yang J, Kim D, Kim B, Roh K, Lee D, Auh C, Lee S (2010). Expression analysis of Proline metabolism-related genes from halophyte Arabis stelleri under osmotic stress conditions. J Integr Plant Biol.

[35] Senthil-Kumar M, Mysore KS (2012). Ornithine-delta-aminotransferase and proline dehydrogenase genes play a role in non-host disease resistance by regulating pyrroline-5-carboxylate metabolism-induced hypersensitive response. Plant Cell Environ.

[36] Lu L, Chen X, Zhu L, Li M, Chen J (2020). NtCIPK9: a Calcineurin B-like protein-interacting protein kinase from the halophyte Nitraria tangutorum, Enhances Arabidopsis Salt Tolerance. Frontiers in Plant ence.

[37] Liu J, Zhu JK (1997). Proline accumulation and salt-stress-induced gene expression in a salt-hypersensitive mutant of Arabidopsis. Plant Physiol.

[38] Verbruggen N, Hermans C (2008). Proline accumulation in plants: a review. Amino Acids.

[39] Bailly C, Benamar A, Corbineau F, Come D (2010). Changes in malondialdehyde content and in superoxide dismutase, catalase and glutathione reductase activities in sunflower seeds as related to deterioration during accelerated aging. Physiol Plant.

[40] Jha B, Sharma A, Mishra A (2011). Expression of SbGSTU (tau class glutathione S-transferase) gene isolated from Salicornia brachiata in tobacco for salt tolerance. Mol Biol Rep.

[41] Yu Y, Xia X, Yin W, Zhang H (2007). Comparative genomic analysis of CIPK gene family in Arabidopsis and Populus. Plant Growth Regul.

[42] Verbruggen N, Hermans C (2008). Proline accumulation in plants: a review. Amino Acids.

[43] Xiang Y, Huang Y, Xiong L (2007). Characterization of stress-responsive CIPK genes in rice for stress tolerance improvement. Plant Physiol.

[44] Teruaki T, Motoaki S, Masakazu S, Tetsuya S, Masatomo K, Kanako I, Yoshihiro N, Mari N, Jian-Kang Z, Kazuo S (2004). Comparative genomics in salt tolerance between Arabidopsis and aRabidopsis-related halophyte salt cress using Arabidopsis microarray. Plant Physiol.

[45] Zhao KF, Harris PJC (1992). The effects of Iso-osmotic salt and water stresses on the growth of halophytes and non-halophytes. J Plant Physiol.

[46] Liu L, Wang Y, Wang N, Dong Y, Fan X, Liu X, Yang J, Li H (2011). Cloning of a vacuolar H+-pyrophosphatase gene from the halophyte Suaeda corniculata whose heterologous overexpression improves salt, saline-alkali and drought tolerance in Arabidopsis. J Integr Plant Biol.

[47] Ben SR, Zouari N, Ben RW, Azaza J, Meynard D, Guiderdoni E, Hassairi A (2010). Improved drought and salt stress tolerance in transgenic tobacco overexpressing a novel A20/AN1 zinc-finger "AlSAP" gene isolated from the halophyte grass Aeluropus littoralis. Plant Mol Biol.

[48] Yao M, Zeng Y, Liu L, Huang Y, Zhao E, Zhang F (2012). Overexpression of the halophyte Kalidium foliatum H^+^-pyrophosphatase gene confers salt and drought tolerance in Arabidopsis thaliana. Mol Biol Rep.

[49] Sanchezbarrena MJ, Martinezripoll M, Zhu J, Albert A (2005). The structure of the Arabidopsis Thaliana SOS3: molecular mechanism of sensing calcium for salt stress response. J Mol Biol.

[50] Li R, Zhang J, Wu G, Wang H, Chen Y, Wei J (2012). HbCIPK2, a novel CBL-interacting protein kinase from halophyte Hordeum brevisubulatum, confers salt and osmotic stress tolerance. Plant Cell Environ.

[51] Miranda RDS, Alvarezpizarro JC, Costa JH, Paula SDO, Prisco JT, Gomesfilho E. Putative role of glutamine in the activation of CBL/CIPK signalling pathways during salt stress in sorghum. Plant Signal Behav. 2017:**12**(8). 10.1080/15592324.2017.1361075 PMC5616156 28805497

[52] Hu D, Ma Q, Sun C, Sun M, You C, Hao Y (2016). Overexpression of MdSOS2L1, a CIPK protein kinase, increases the antioxidant metabolites to enhance salt tolerance in apple and tomato. Physiol Plant.

[53] Hare PD, Cress WA, Van Staden J (1998). Dissecting the roles of osmolyte accumulation during stress. Plant Cell and Environment.

[54] Abdula SE, Lee HJ, Ryu H, Kang KK, Nou I, Sorrells ME, Cho Y (2016). Overexpression of BrCIPK1 gene enhances abiotic stress tolerance by increasing Proline biosynthesis in Rice. Plant Mol Biol Report.

[55] Zheng L, Dang Z, Li H, Zhang H, Wu S, Wang Y (2014). Isolation and characterization of a Δ1-pyrroline-5-carboxylate synthetase (NtP5CS) from Nitraria tangutorum Bobr. And functional comparison with its Arabidopsis homologue. Mol Biol Rep.

[56] Luan S (2009). The CBL-CIPK network in plant calcium signaling. Trends in Plant ence.

[57] Gadallah MAA (1999). Effects of Proline and Glycinebetaine on Vicia Faba responses to salt stress. Biol Plant.

[58] Hellebusi JA (1976). Osmoregulation. Plant Biol.

[59] Smirnoff N, Cumbes QJ (1989). Hydroxyl radical scavenging activity of compatible solutes. Phytochemistry.

[60] Misra N, Gupta AK (2005). Effect of salt stress on proline metabolism in two high yielding genotypes of green gram. Plant Sci.

[61] Ma Y, Cao J, Chen Q, He J, Liu Z, Wang J, Li X, Yang Y (2019). The kinase CIPK11 functions as a negative regulator in drought stress response in Arabidopsis. Int J Mol Sci.

[62] Clough SJ, Bent AF (2010). Floral dip: a simplified method for agrobacterium-mediated transformation of Arabidopsis thaliana. Plant J.

[63] Janmohammadi M, Abbasi A, Sabaghnia N. Influence of NaCl treatments on growth and biochemical parameters of castor bean (*Ricinus communis* L.). Acta Agriculturae Slovenica. 2012:99(1).

[64] Zhou G, Nimir NEA, Lu S, Zhai F, Wang Y (2014). Gibberellic acid and salinity affected growth and antioxidant enzyme activities in Castor bean plants at early growth stage. Agron J.

[65] Li W, Fei-Feng LI, Wen-Bo Z, Gui-Lin C, Xiao-Fei L (2012). Isolation and characterization of Nitraria sibirica actin gene. Acta Pratacul Sin.

[66] Norris SR, Meyer SE, Callis J (1993). The intron of Arabidopsis thaliana polyubiquitin genes is conserved in location and is a quantitative determinant of chimeric gene expression. Plant Mol Biol.

[67] Thordal-Christensen H, Zhang Z, Wei Y, Collinge DB. Subcellular localization of H2O2 in plants. H2O2 accumulation in papillae and hypersensitive response during the barley—powdery mildew interaction. Plant J. 1997:11.

