# Supplementary figures and images for: CIPK11: a calcineurin B-like protein-interacting protein kinase from Nitraria tangutorum, confers tolerance to salt and drought in Arabidopsis
Source: BMC Plant Biol. 2021 Mar 1;21:123. doi: 10.1186/s12870-021-02878-x (PMC7919098; doi:10.1186/s12870-021-02878-x)

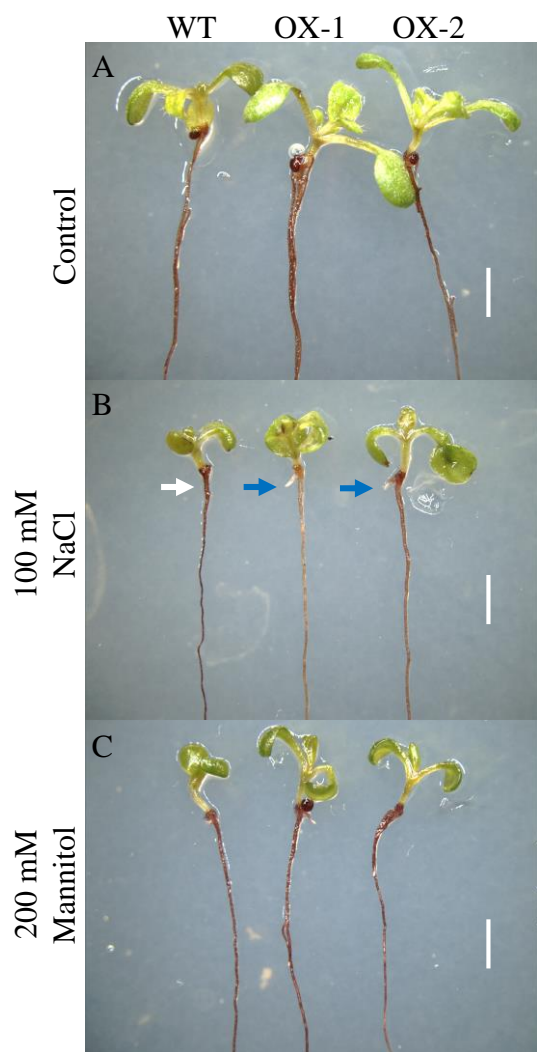

Supplement: Supplementary file 2 — Additional file 2: Fig. S1. Detection of H2O2 accumulation in Arabidopsis. H2O2 staining with DAB for one-week-old Arabidopsis seedlings (left: WT; middle: OX-1; right: OX-2) cultured under the control condition (A), 100 mM NaCl treatment (B) and 200 mM Mannitol treatment (C). Blue arrows show the light brown root; white arrow shows the dark brown root. Scale bar: 0.2 cm. [file 12870_2021_2878_MOESM2_ESM.pdf]
